# ENO1‐targeted superparamagnetic iron oxide nanoparticles for detecting pancreatic cancer by magnetic resonance imaging

**DOI:** 10.1111/jcmm.15237

**Published:** 2020-04-13

**Authors:** Lei Wang, Hang Yin, Rongrong Bi, Guo Gao, Kaicheng Li, Hai‐Lin Liu

**Affiliations:** ^1^ Department of Gastroenterology The Ninth People's Hospital Affiliated to the School of Medicine Shanghai Jiaotong University Shanghai China; ^2^ Department of Pulmonary Longhua Hospital Shanghai University of Traditional Chinese Medicine Shanghai China; ^3^ Department of Instrument Science and Engineering Key Laboratory for Thin Film and Microfabrication Technology of the Ministry of Education Institute of Nano Biomedicine and Engineering Shanghai Jiao Tong University Shanghai China; ^4^ Department of Radiology The Ninth People's Hospital Affiliated to the School of Medicine Shanghai Jiaotong University Shanghai China

**Keywords:** diagnosis, molecular imaging, pancreatic neoplasms, phosphopyruvate hydratase

## Abstract

The aim of this study was to investigate in vitro magnetic resonance imaging (MRI) of PDAC using ENO1‐targeted superparamagnetic iron oxide nanoparticles and xenograft models. Expression level and location of ENO1 protein in pancreatic cancer cell lines of CFPAC‐1 and MiaPaCa‐2 were detected by Western blotting, flow cytometry and confocal microscopy. Dex‐g‐PCL/SPIO nanoparticles targeting ENO1 were constructed with ENO1 antibody and characterized by MRI. In addition, ENO1‐Dex‐g‐PCL/SPIO nanoparticles were tested to assess their efficacy on the detection of PDAC using in vitro and in vivo MRI. The results showed that ENO1 was expressed in both human PDAC cell lines of CFPAC‐1 and MiaPaCa‐2, demonstrating that the localization of cytoplasm and membrane was dominant. It was confirmed that ENO1 antibody was connected to the SPIO surface in ENO1‐Dex‐g‐PCL/SPIO nanoparticles. The nanoparticles had satisfactory superparamagnetism and significantly enhance the detection of PDAC by in vivo and in vitro MRI. In conclusion, ENO1 can serve as a membrane protein expressed on human PDAC cell lines. ENO1‐targeted SPIO nanoparticles using ENO1 antibody can increase the efficiency of detection of PDAC by in vitro and in vivo MRI.

## 1 INTRODUCTION

Pancreatic ductal adenocarcinomas (PDACs) are characterized by delayed onset, rapid progression and high metastasis. PDACs are one of the most fatal forms of human cancer with a five‐year survival rate of 5% for all stages.^1^, ^2^ This poor prognosis is related to the difficulty in early diagnosis and the late stages of the disease stage at the time of diagnosis, reflecting the necessity for early detection of new approaches of PDAC.

Molecular imaging, defined as the characterization and measurement of biological processes at the cellular and molecular levels, has significantly attracted scholars' attention in the detection of malignant diseases. Combining magnetic resonance imaging with advanced contrast agents, it is possible to perform molecular analysis of the targeted histiocytosis and make early detection of cancer possible. Superparamagnetic iron oxide (SPIO) has been widely applied in MRI as a contrast agent, and conjugation of SPIO with specific ligands, such as antibodies, peptides and nucleotides, can result in the appearance of a variety of targeted SPIO nanoparticles.^3^, ^4^, ^5^, ^6^, ^7^ Targeted SPIO nanoparticles are ideal carriers for the fabrication of imaging probes with their own characteristics and the realization of PDAC molecular imaging.

Enolase 1 (ENO1), also named as pyruvate dehydrogenase 1, is a glycolytic enzyme, acting as a multifunctional moonlight protein.^8^, ^9^ It is up‐regulated at the mRNA/protein level in PDAC cell lines and tissues and is involved in development, invasion, metastasis and chemoresistance of PDAC.^10^, ^11^ ENO1 can be a cytoplasmic protein with enzymatic activity and be translated into C‐Myc promoter‐binding protein (MBP‐1) in the nucleus^12^; furthermore, ENO1 can serve as a membrane protein expressed on the cell membrane, acting as a plasminogen receptor.^13^, ^14^ Additionally, ENO1 located on cell membrane of PDAC might be beneficial for magnetic resonance molecular imaging with SPIO nanoparticles.

In the present study, we constructed ENO1‐targeted Dex‐g‐PCL/SPIO nanoparticles with ENO1 antibody. The ability of ENO1‐targeted SPIO nanoparticles in detecting pancreatic cancer by MRI was assessed in both in vitro and in vivo experiments. The successful construction of ENO1‐targeted SPIO nanoparticles would facilitate early and accurate detection PDACs, paving the way for pancreatic cancer treatment. This original method of constructing nanoparticles provided new approaches for targeting cancer cells, which might also be used in the early diagnosis of other diseases.

## 2 MATERIALS AND METHODS

### 2.1 Cell lines

Human PDAC cell lines of CFPAC‐1 and MiaPaCa‐2 were purchased from American Type Culture Collection (Manassas, VA, ATCC). MiaPaCa‐2 cells were cultured in Dulbecco's modified Eagle's medium (DMEM; HyClone Laboratories, Inc), and CFPAC‐1 cells were cultured in Roswell Park Memorial Institute (RPMI)‐1640 medium (HyClone Laboratories, Inc), both supplemented with 10% foetal bovine serum (FBS; Gibco), and 0.01% penicillin‐streptomycin at 37°C in an atmosphere of 21% O_2_ and 5% CO_2_.

### 2.2 Western blot analysis

To determine the expression level of ENO1 protein in CFPAC‐1 and MiaPaCa‐2 cells, the cells (1 × 10^7^) were harvested for Western blot analysis. In addition, 20 μg of total protein was separated by sodium dodecyl sulphate‐polyacrylamide gel electrophoresis (SDS‐PAGE), followed by transferring into nitrocellulose filter (NC) membranes (EMD Millipore) using a semi‐dry electrophoretic transfer cell system (Bio‐Rad Laboratories, Inc). The membranes were blocked with 5% non‐fat milk for 1 hour and then incubated with anti‐ENO1 (Abcam) or an antibody specific to glyceraldehyde 3‐phosphate dehydrogenase (GAPDH) (Sigma‐Aldrich Corp.) overnight at 4°C. The membranes were then incubated with horseradish peroxidase (HRP)–conjugated secondary antibodies (Santa Cruz Biotechnology, Inc) for 1 hour at room temperature. Blots were detected with enhanced chemiluminescence (ECL) reagents (EMD Millipore) and exposed to a chemiluminescent imaging system for 5 minutes.

### 2.3 Flow cytometry analysis

Here, PDAC cells of CFPAC‐1 and MiaPaCa‐2 were spun down and re‐suspended in 500‐µL phosphate‐buffered saline (PBS), in which cells were incubated with a primary anti‐ENO1 (Abcam) or an isotype‐matched negative control antibody for 30 minutes at 4°C, and the analysis was conducted using the FACScan flow cytometer (BD Biosciences).

### 2.4 Immunofluorescence analysis

To determine the cellular location of ENO1 protein in CFPAC‐1 and MiaPaCa‐2 cells, PDAC cells were incubated with primary antibody, and anti‐ENO1 (Abcam) diluted in 1% bovine serum albumin (BSA) (1:200) overnight at 4°C. Coverslips were washed and incubated with secondary antibody, FITC‐goat anti‐rabbit (1:50) for 20 minutes at 37°C. Coverslips were washed; fixed for 20 minutes in 4% paraformaldehyde, 0.25‐mmol/L glycine for 20 minutes at 37°C; and mounted onto glass slides. Images were taken using a Zeiss LSM 510 Meta confocal microscope (Carl Zeiss AG).

### 2.5 Construction and characterization of ENO1‐targeted Dex‐g‐PCL/SPIO nanoparticles

The first is to prepare SPIO nanoparticles, in which 1 mmol Fe(acac)3 with 5 mmol 1,2‐hexadecanol, 3 mmol oleic acid and 3 mmol oleylamine were mixed in 10 mL benzyl ether, heated to 300°C for 1 hour under argon protection. The solution was precipitated with methanol at room temperature, and the obtained black solid product was kept in n‐hexane. Next, poly(epsilon‐caprolactone)‐grafted dextran (Dex‐g‐PCL) was prepared as previously reported.^15^ Dex‐g‐PCL/SPIO nanoparticles in the aqueous phase were prepared through the emulsion evaporation method. Next, 50 µL of Enolase‐1 monoclonal antibody (0.5 mg/mL, Abcam) was mixed to the Dex‐g‐PCL/SPIO nanoparticles solution at room temperature for 15 minutes, and 2 mg of carbodiimide was added at room temperature for 30 minutes. The sample was transferred to a dialysis bag and dialysed for 24 hours in PBS solution at 4°C. Afterwards, suspension of ENO1‐Dex‐g‐PCL/SPIO nanoparticles was taken out from the dialysis bag and stored at 4°C until use. The size of the nanoparticles was examined using a transmission electron microscope (Joel, JEM‐1011). The diameters of nanoparticles were measured by Malvern Zetasizer (Malvern Panalytical Ltd.). The nanoparticles were also detected with X‐ray diffractometer (Jordan Valley Semiconductors, Ltd.) and Fourier infrared spectroscopy (Fairborn, China). The binding specificity of ENO1‐Dex‐g‐PCL/SPIO nanoparticles to their targets in cells was examined through Prussian blue staining after cultivating for 6 hours.

### 2.6 Magnetic property and T2 relativity analysis

Magnetic properties of ENO1‐Dex‐g‐PCL/SPIO nanoparticles were detected by superconducting quantum interference magnetometer (Quantum Design Inc) at room temperature after lyophilization. Next, the efficiency of T2 relaxation for nanoparticles was examined by 1.5T MRI (Siemens Magnetom Trio Tim, Siemens AG) as previously reported.^16^

We prepared SPIO and ENO1‐SPIO granule solutions with iron concentrations of 30, 15, 7.5, 3.75, 1.875 and 0.9375 ug/mL and then added 1 mL 2% agarose to prepare a final concentration of 1% agarose. Next, 1% agarose was used as a blank control to detect the T2 value and T2* value of different concentrations of iron particle solution. The corresponding relaxation rates R2 and R2* were calculated as 1/T2 and 1/T2*, respectively.

### 2.7 In vitro MRI

Here, CFPAC‐1 and MiaPaCa‐2 cells were plated at a density of 3 × 10^5^ cells/well in a 24‐well plate and then treated with 10‐ul SPIO (Fe with concentration of 0.1 mg Fe/mL), ENO1‐SPIO, and PBS control for 2 hours, and scanned for MRI as well.

### 2.8 Pancreatic cancer xenograft model and in vitro MRI

In this study, 12 male BALB/c nude mice (age, 7‐week‐old; weight, 20‐25 g) were kept in specific pathogen‐free (SPF) facilities. After that, 2 × 10^7^ CFPAC‐1 cells were subcutaneously injected for about 3 weeks as previously reported.^17^ When the tumours grew to about 10 mm diameter in size, the tumour would be appropriate for MRI. The animal research protocol used here was approved by the Animal Farewell Committee of the Ninth People's Hospital Affiliated to Shanghai Jiao Tong University School of Medicine (Shanghai, China).

Tumour‐bearing nude mice scanning was performed on a 1.5T MR scanner (Siemens Magnetom Trio Tim; Semens AG) using an animal coil. The mice were anaesthetized using Ketamine (4.0 mg/100 g). Mice were scanned in advance, and 2, 4, 12 and 24 hours after injection with the SPIO or ENO1‐SPIO (body weight, 0.1 mL/20 g) through the tail vein.

The imaging sequences are as follows: TR of 4200 ms and TE of 36 ms were used for T2‐weighted fast‐spin‐echo imaging, a field of view (FOV) of 40 mm × 40 mm and slice thickness of 1 mm. Region of interest (ROI) was utilized for signal measurement, and 20‐mm^2^ field at the largest level of tumour imaging was selected as ROI.

### 2.9 Histologic analysis

Mice were killed in a CO_2_ chamber after MRI. Tissues were collected and kept in formaldehyde solution for 24 hours at 4°C, and then embedded in paraffin. Tissue sections were stained with haematoxylin and eosin (H&E) for histology or Prussian blue to detect blue iron nanoparticles. Immunohistochemistry (IHC) of the tissues was carried out according to the previously reported protocols.^11^

### 2.10 Statistical analysis

Data were expressed as mean ± standard deviation (SD). Differences between two groups were analysed by the Student's *t* test. To analyse differences among three or more groups, one‐way analysis of variance (ANOVA) was utilized. The statistical analysis was undertaken by GraphPad Prism 6 software; *P*‐values less than 0.05 were statistically considered significant.

## 3 RESULTS

### 3.1 ENO1 expression of pancreatic cancer cell lines

**FIGURE 1:**
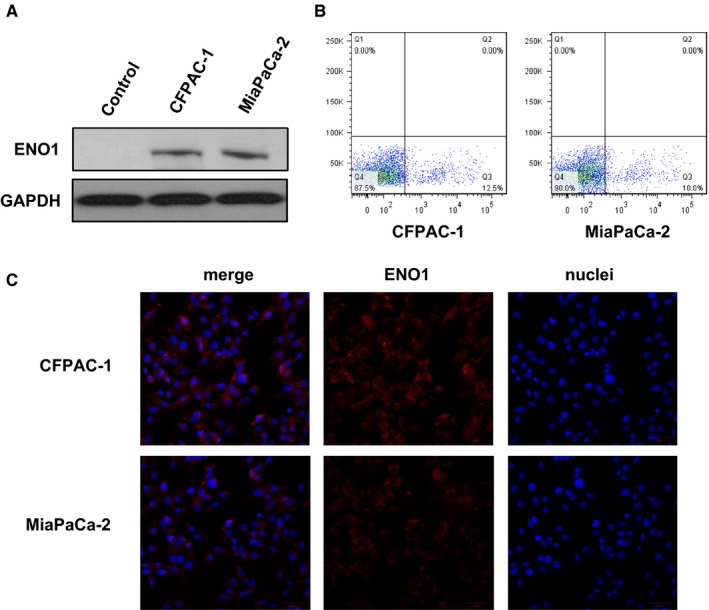
ENO1 expression of pancreatic cancer cell lines. A, Total expression level of ENO1 protein in MiaPaCa‐2 and CFPAC‐1 cells detected by Western blotting. B, Representative images showed cell‐surface expression of ENO1, which was analysed by flow cytometry in MiaPaCa‐2 and CFPAC‐1 cells. C, Subcellular expression of ENO1 was detected by immunofluorescence analysis in MiaPaCa‐2 and CFPAC‐1 cells. Results were achieved from representative experiments in triplicate

To clarify the expression of ENO1 and the location of pancreatic cancer lines, CFPAC‐1 and MiaPaCa‐2 cells were analysed by Western blotting, flow cytometry and immunofluorescence staining. Results of Western blotting showed that ENO1 was expressed in both MiaPaCa‐2 and CFPAC‐1 cells (Figure 1A). Flow cytometry, using ENO1 antibody, further confirmed the cell‐surface expression of pancreatic cell lines (Figure 1B). Immunofluorescence analysis confirmed that ENO1 displayed a predominant cytoplasm and membrane localization (Figure 1C). These results suggested that ENO1 is a protein target, which might be used in magnetic resonance molecular imaging with SPIO nanoparticles.

### 3.2 Characterization of ENO1‐Dex‐g‐PCL/SPIO nanoparticles

**FIGURE 2:**
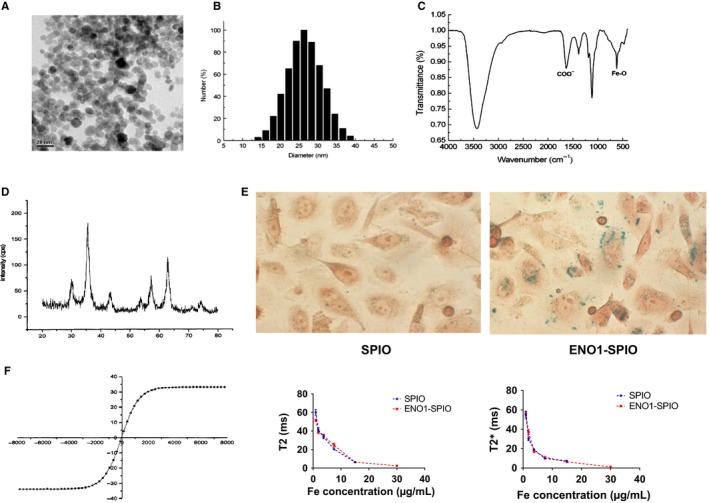
Characterization of ENO1‐Dex‐g‐PCL/SPIO nanoparticles. A and B, ENO1‐Dex‐g‐PCL/SPIO nanoparticles had an average total size of 30 nm. C, The nanoparticles were detected by Fourier transform infrared spectroscopy, and the characteristic C = O absorption peak demonstrated that ENO1 antibody was connected to the SPIO surface. D, X‐ray diffractometry indicated the sample was mainly consisted of Fe_3_O_4_ with complete crystal structure. E, Prussian blue staining showed more blue‐stained particles incubated with ENO1‐SPIO in CFPAC‐1 cells compared with SPIO. F, The hysteresis curve demonstrated that the nanoparticles had an appropriate property of superparamagnetism, and significantly decreases of T2 and T2* relaxation time were detected at different iron concentrations. Results were achieved from representative experiments in triplicate

ENO1‐Dex‐g‐PCL/SPIO nanoparticles had a Fe_3_O_4_ core size of 5‐10 nm and average total size of 30 nm. The majority of the SPIO particles were dispersed, and a small number of particles were aggregated (Figure 2A,B).

The nanoparticles were detected by Fourier transform infrared spectroscopy. The characteristic of C = O absorption peak appeared in the absorption spectrum, indicating that the amide bond was formed by the carbodiimide method between the carboxyl group located at the surface of the particle and the amino group of ENO1 antibody, which confirmed that ENO1 antibody was connected to the SPIO surface (Figure 2C).

In addition, X‐ray diffractometry revealed that the diffraction line of the complex had seven characteristic peaks at 2θ angles, which were located at 30.05, 35.59, 43.21, 53.62, 57.23, 62.77 and 74.15. The position and relative intensity of the peak indicated that the sample was mainly consisted of Fe_3_O_4_ with the complete crystal structure (Figure 2D).

Prussian blue staining showed more blue‐stained particles in CFPAC‐1 cells incubated with ENO1‐Dex‐g‐PCL/SPIO nanoparticles, demonstrating that the absorption of antibody‐modified polymer nanoparticles depends on the binding of antibody ligands (Figure 2E).

The hysteresis curve demonstrated that ENO1‐Dex‐g‐PCL/SPIO nanoparticles had a satisfactory property of superparamagnetism (Figure 2F). Significant decreases of T2 and T2* relaxation time were detected at different iron concentrations with SPIO and ENO1‐SPIO.

### 3.3 In vitro MRI of ENO1‐Dex‐g‐PCL/SPIO nanoparticles

**FIGURE 3:**
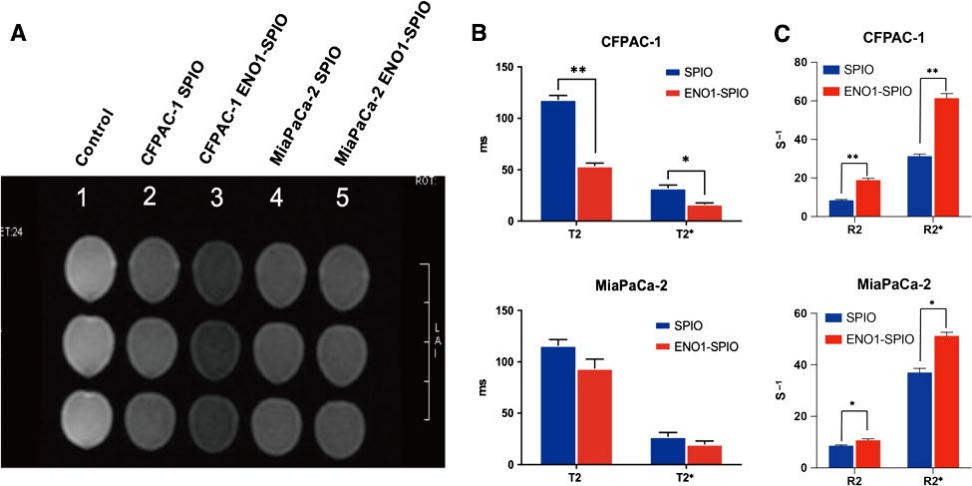
In vitro MRI of ENO1‐Dex‐g‐PCL/SPIO nanoparticles. A, MRI of ENO1‐Dex‐g‐PCL/SPIO nanoparticles in cultured CFPAC‐1 and MiaPaCa‐2 cells. B, Compared with SPIO group, the T2 and T2* values of CFPAC‐1 cells were notably decreased, which were incubated with ENO1‐SPIO. C, The R2 and R2* values of CFPAC‐1 cells were notably increased in ENO1‐SPIO group compared with SPIO group. Results were achieved from representative experiments in triplicate and were shown as mean ± standard deviation (SD). *P < .05, **P < .01

The findings disclosed that compared with the SPIO group, the T2 and T2* values of CFPAC‐1 and MiaPaCa‐2 cells were decreased after incubating with ENO1‐SPIO, and the T2 and T2* values of CFPAC‐1 cells were significant (Figure 3A,B). Similarly, significant increase of R2 and R2* value was observed after incubating with ENO1‐SPIO. The R2/R2* value was 0.27 for SPIO and 0.30 for ENO1‐SPIO in CFPAC‐1, higher than 0.23 for SPIO and 0.21 for ENO1‐SPIO in MiaPaCa‐2.

### 3.4 In vivo MRI of ENO1‐Dex‐g‐PCL/SPIO nanoparticles

**FIGURE 4:**
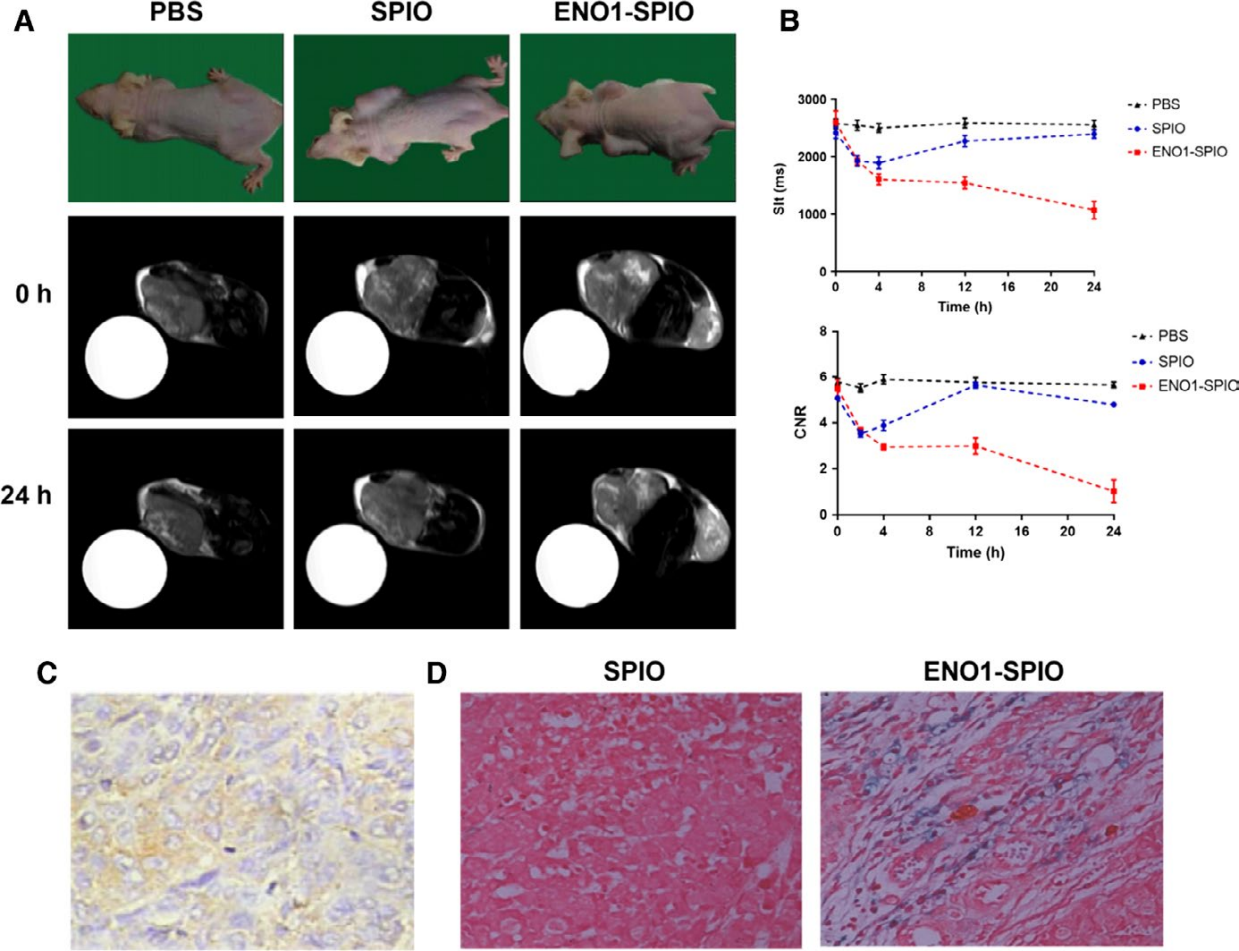
Detection of pancreatic tumour by in vivo MRI of ENO1‐Dex‐g‐PCL/SPIO nanoparticles. A, MRI of ENO1‐Dex‐g‐PCL/SPIO nanoparticles in a pancreatic cancer xenograft model. B, Compared with PBS control group and SPIO group, the T2 signal intensity of tumour tissue significantly decreased, and the tumour gradually darkened over time, in which the peak of enhancement was at 24 h in ENO1‐SPIO group. C, IHC staining (40×) of the pancreatic tumour tissues 24 h after injection with ENO1‐SPIO nanoparticles. D, Prussian blue staining (40×) of the pancreatic tumour tissues 24 h after injection with ENO1‐SPIO or SPIO. More positive iron particles were found in ENO1‐SPIO group. Results were achieved from representative experiments in triplicate and were shown as mean ± standard deviation (SD). *P < .05, **P < .01

The results showed that the tissues had equal and higher signal on the T2 sequence during MRI, the signal was not uniform, and necrosis was observed inside the tumour (Figure 4A). After non‐targeted contrast agent SPIO was injected into the control group, the signal intensity gradually increased 1 hour after the early non‐specific enhancement, which reached the peak after 2 hours, and was fully recovered to the pre‐injection level after 24 hours. After the injection of ENO1‐SPIO targeting contrast agent, the T2 signal intensity of tumour tissue significantly decreased, and the tumour gradually darkened over time, and the peak of enhancement was observed at 24 hours (Figure 4B). IHC staining demonstrated ENO1 expression in pancreatic tumour tissues of xenograft model (Figure 4C). Prussian blue staining revealed that more positive iron particles were found in ENO1‐SPIO group compared with SPIO group (Figure 4D).

## 4 DISCUSSION

Poor prognosis of PDAC indicates the necessity of discovering new approaches for early detection, and molecular imaging is an emerging attractive method to detect malignant diseases including PDAC. Among all the major imaging modalities contributing to molecular imaging, MRI is particularly attractive because it allows for molecular profiling of target tissues/cells with its superior spatial resolution with an advanced contrast agent.

In recent years, SPIO has been conjugated with specific ligands, such as antibodies, peptides, polysaccharides, nucleotides, aptamers and other synthetic mimetics to create a variety of targeted SPIO,^5^, ^6^, ^7^, ^18^, ^19^, ^20^, ^21^ which could be used as contrast agents and have been extensively applied in the magnetic resonance molecular imaging. However, the sensitivity and specificity of these targets for PDAC diagnosis remain unsatisfactory. Montet et al^22^ performed imaging of PDAC using functional SPIO targeted to bombesin (BN) receptor present on the normal acinar cells of the pancreas. The BN peptide‐nanoparticle conjugate, BN‐CLIO (Cy5.5), decreased the T2 of normal pancreas and enhanced the ability to visualize tumour in a model of pancreatic cancer by MRI. Kelly et al^23^ demonstrated that plectin‐1‐targeted peptides conjugated to SPIO enabled detection of small PDAC and precursor lesions in engineered mouse models. Yang et al^24^ reported that the systemic delivery of urokinase‐type plasminogen activator receptor (uPAR)‐targeted SPIO resulted in selective accumulation within tumours of an orthotopic human pancreatic cancer xenograft model in nude mice. Therefore, it is urgent to find out new cell‐surface targets for SPIO to improve the diagnosis of PDAC.

ENO1, as a key glycolytic enzyme involved in catalysing the conversion of 2‐phospho‐D‐glycerate to phosphoenolpyruvate, can enhance glycolytic process in tumour cells because of the Warburg effect.^25^, ^26^ Studies revealed that ENO1 belongs to a group of moonlight protein, which plays a key role in many biological and pathophysiological processes.^27^ In the majority of cases, ENO1 is a cytoplasmic protein with enzymatic activity, and ENO1 can also serve as a membrane protein expressed on cell membrane, acting as a plasminogen receptor promoting cell invasion; besides, ENO1 can be translated into MBP‐1 in the nucleus, leading to tumour suppression.^12^ Previous studies showed that ENO1 located on the surface in tumour cells is subjected to post‐translational modifications, including acetylation, methylation and phosphorylation.^9^, ^28^, ^29^ It was also proved that the expression of ENO1 was not up‐regulated in chronic pancreatitis, similar to normal pancreas tissues.^8^ Our results confirmed that ENO1 is expressed on the cell surface of two pancreatic cell lines; thus, ENO1 might have a superior surface molecular characteristic for magnetic resonance molecular imaging using SPIO nanoparticles.

In the present study, our results indicated that ENO1 can serve as a membrane protein expressed on PDAC cell lines, and we conjugated SPIO with ENO1 antibody to develop ENO1‐Dex‐g‐PCL/SPIO nanoparticles, which appropriate property of superparamagnetism. The specific accumulation of ENO1‐Dex‐g‐PCL/SPIO nanoparticles is based on the receptor‐mediated uptake of nanoparticles into targeted cells (intracellular trapping). Our results demonstrated that ENO1‐Dex‐g‐PCL/SPIO nanoparticles could increase the efficacy of detecting PDAC by in vivo and in vitro MRI. These results suggested that ENO1 might be a selected target for molecular imaging by MRI.

## 5 CONCLUSIONS

ENO1 can serve as a membrane protein expressed on human PDAC cell lines. ENO1‐targeted SPIO nanoparticles using ENO1 antibody can increase the efficiency of detection of PDAC by in vitro and in vivo MRI.

## CONFLICT OF INTEREST

All authors declare that they have no conflict of interest.

## AUTHORS' CONTRIBUTIONS

Lei Wang and Hai‐Lin Liu participated in experimental design. Lei Wang, Hang Yin, Guo Gao, Kaicheng Li and Rongrong Bi carried out the experiments. Lei Wang and Hang Yin participated in data interpretation and manuscript preparation.

## Data Availability

Data were available on request from the corresponding author.
